# Two‐Photon 3D Printing of Functional Microstructures Inside Living Cells

**DOI:** 10.1002/adma.202519286

**Published:** 2026-01-14

**Authors:** Maruša Mur, Aljaž Kavčič, Uroš Jagodič, Rok Podlipec, Matjaž Humar

**Affiliations:** ^1^ Department of Condensed Matter Physics J. Stefan Institute Ljubljana Slovenia; ^2^ Faculty of Mathematics and Physics University of Ljubljana Ljubljana Slovenia; ^3^ CENN Nanocenter Ljubljana Slovenia

**Keywords:** 3D printing, intracellular biofabrication, intracellular devices, two‐photon lithography

## Abstract

3D printing is transforming manufacturing and biomedicine, yet it has not been demonstrated inside living cells. Additionally, there is no method to deliver micron‐scale, free‐standing solid microstructures directly into the cytosol of non‐phagocytic cells. Here, both of these challenges are addressed by fabricating custom‐shaped polymeric microstructures directly inside living cells using two‐photon polymerization. A bio‐compatible photoresist is injected into cells and selectively polymerized with a femtosecond laser, creating intracellular structures with submicron resolution. Structures of various shapes are printed in live cells, including a 10 μm elephant, barcodes for cell tracking, diffraction gratings for remote readout, and microlasers. The printed structures in cells can affect the cell biology. The demonstrated top‐down intracellular biofabrication approach, combined with functional photoresists, may enable new applications in intracellular sensing, biomechanical manipulation, bioelectronics, and targeted drug delivery. These embedded structures could provide novel control over the intracellular environment, allowing engineering of cellular properties beyond natural limits and genetic engineering.

## Graphical abstract

This study demonstrates 3D printing inside living cells by using two‐photon photo‐lithography. A bio‐compatible photoresist is injected into cells and selectively polymerized with a femtosecond laser, creating custom‐shaped intracellular structures with submicron resolution. The paper discusses the behavior of cells containing the printed structures, the fidelity of the 3D‐printed structures, and possible applications of the demonstrated method.

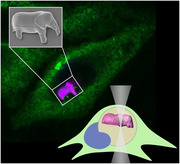

## 1 Introduction

Over the past decade, 3D printing has become an indispensable tool in industry and various scientific fields, such as electronics [1], soft robotics [2], micro‐optics and photonics [3], biology [4], and biomedicine [5]. For nano‐ and micro‐scale structures, various 3D printing techniques can be used. The best printing resolution can be achieved with the two‐photon polymerization (TPP) technique [6], where a photo‐sensitive resin (a photoresist) is illuminated with a femtosecond laser. Two‐photon absorption occurs only in the small volume of the laser‐beam focus, where the laser intensity is sufficiently high. This results in the photo‐polymerization occurring only in a limited voxel, enabling printing features that are down to 100 nm in size.

In biological environments, TPP is performed with bio‐compatible photoresists, mostly to print cellular scaffolds and for tissue regeneration [4]. Cells or bacteria can be added to the photoresist to print cellular structures of custom shapes [7, 8]. Lately, TPP 3D printing has been demonstrated in living organisms. In one case, a photo‐responsive hydrogel ink was injected into mouse tissues to print ∼ 1 mm structures [9]. In another example, objects 50–60 μm in diameter were printed by TPP inside a live embryo of a fruit fly or of a medaka fish [10].

There has been one report on TPP inside a synthetic cell [11], however, we have not found any documented attempt at 3D printing objects inside living cells. Instead, for different intracellular applications, micro‐scale objects are typically embedded into cells through various delivery methods, such as endocytosis, phagocytosis, microinjection, and membrane poration. Objects larger than about one micrometer can only be internalized through phagocytosis, which restricts this to cells with phagocytic ability, and even then, they are not delivered directly into the cytosol. Therefore, there is no universal method for delivering larger objects into a cell.

Objects delivered into cells can serve as probes for intracellular microrheology and force measurement with optical [12] or magnetic tweezers [13], and as sensors for various parameters inside the cell (such as refractive index, pH, temperature, etc.) [14, 15, 16]. Another application is barcoding, where a foreign 3D object is used as an identification tag for a cell it resides in. By tagging them, the behavior of individual cells can be studied instead of the usual average responses obtained from large cell populations. Tagging and tracking of cells has been reported with micro‐particles of different shapes acting as either graphical [17] or spectral barcodes (microlasers) [18, 19, 20].

Here, we demonstrate 3D printing directly inside living cells, laying the groundwork for a new class of intracellular bioengineering tools and applications.

## 2 Printing in Living Cells

**FIGURE 1:**
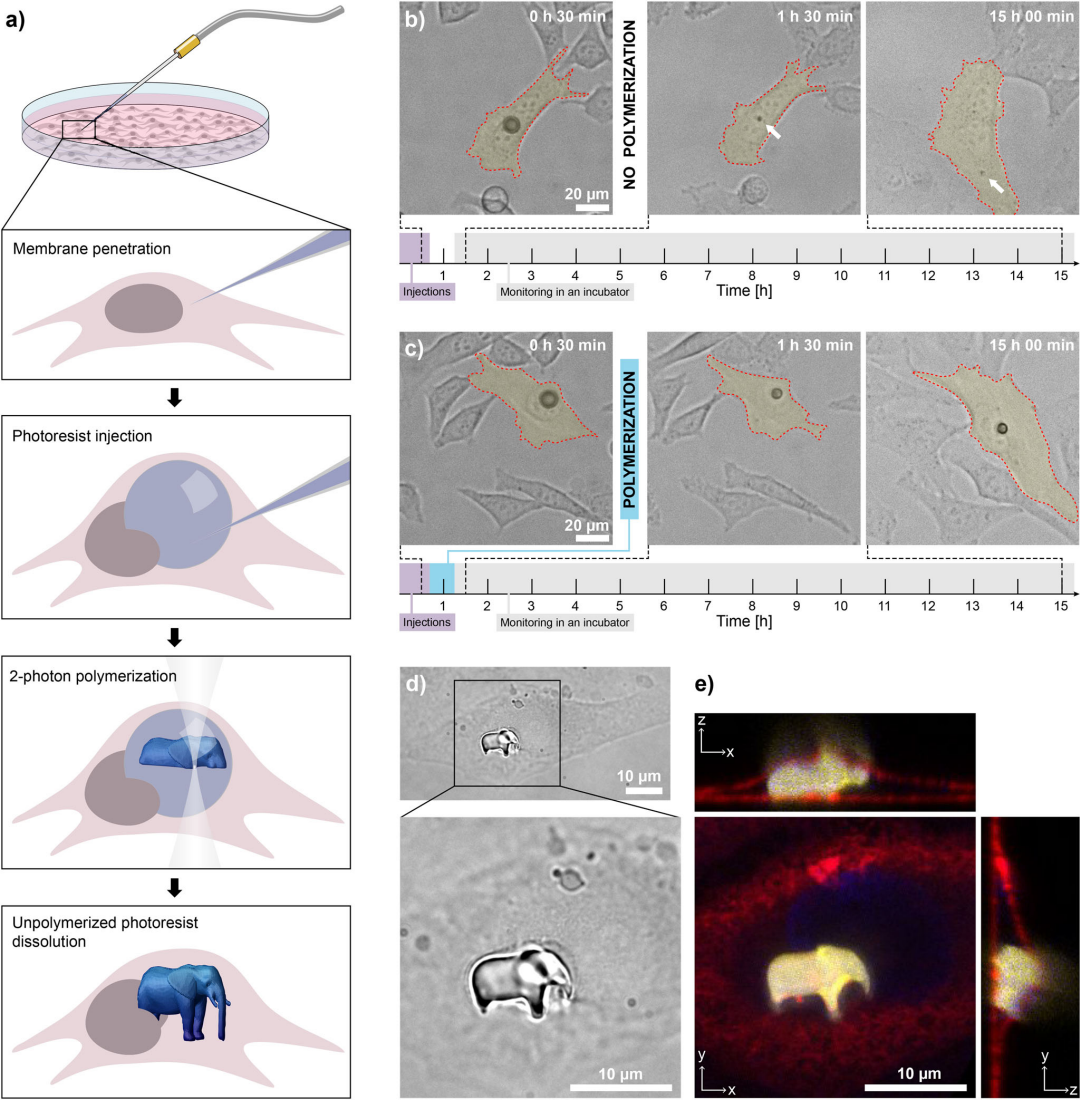
Procedure of 3D printing in cells. (a) Schematic representation of the printing protocol. The photoresist is injected into a cell and illuminated in a pre‐designed pattern to form a polymerized structure. (b) A droplet of photoresist, injected into a live HeLa cell, dissolves almost completely in the first two hours after the injection. White arrows point to the photoresist residue ∼ 2 μm in size, which can sometimes be observed in cells even after a long time. (c) A droplet of photoresist, injected into a live HeLa cell, is completely exposed to laser light ∼ 30 min after the injection. After exposure, the photoresist polymerizes, and the droplet stops dissolving. (d) Bright‐field image of a 10 μm elephant, printed inside a live HeLa cell. (e) Confocal image of the structure in (d). Cross‐sections xz and yz clearly show that the structure is embedded in the cell, as the membrane (red) is seen covering the elephant (yellow). To improve the structure shape recognition and nucleus visibility in the xy‐panel, instead of the usual confocal cross‐sections the yellow and blue channels show maximum‐intensity projections along the z‐axis.

To print inside cells, a droplet of a negative‐tone photoresist was first injected into a live HeLa cell (Figure 1a; Figure S1). The droplet was illuminated in a designed pattern using a commercial system for TPP that incorporates a femtosecond 780 nm laser. The cell as well as the medium are transparent for the near‐IR laser light. Only in the laser focal spot was the light intensity high enough for the material to be polymerized through the process of TPP. The focal spot was moved layer by layer along the designed path, forming a solid structure of a desired 3D shape inside the cell. The unpolymerized photoresist slowly dissolved, leaving only the polymerized structure in the cell. A more detailed experimental timeline is given in Figure S2.

The first step of the study was to find a photoresist that is not toxic to the cells. Several commercially available photoresists are reported to be bio‐compatible when polymerized. However, in our case, the photoresist had to be bio‐compatible also in the unpolymerized state. Additionally, it had to be slightly soluble in water, so that the unpolymerized part could be later slowly dissolved. Out of several commercial photoresists, we chose the IP‐S (Nanoscribe GmbH) as it impacted the cells the least, while also being slightly soluble in an aqueous medium. A droplet approximately 10 μm in diameter dissolves entirely (except for a sometimes observed small photoresist residue) in a few hours in an aqueous environment (Figure 1b). A slight disadvantage of the photoresist solubility is the time limit it sets for the printing: the structures need to be printed within 1–2 h after the photoresist droplet is injected, depending on the initial droplet size. Figure 1c shows an example where a whole droplet was polymerized. After the injection, the droplet is decreasing in size until the laser illumination takes place (between the left and middle panels). After that, the droplet diameter remains constant even after a long time.

After the droplets have been injected into the cells, the two‐photon 3D printing is relatively straightforward and nearly the same as the standard TPP in bulk. The most important difference is that the printed structure has to be aligned with the droplet. The alignment in the plane is straightforward, since the droplet's center can be determined from the microscope image. In the vertical direction, the droplets are held down by the cells, so that the bottom surface of the droplet is practically in contact with the glass substrate. This enables the alignment to the droplet center also in the vertical direction.

In droplets 10–15 μm in diameter, embedded in cells, we printed various 3D structures approximately 10 μm in size (Figure S3). For example, Figure 1d shows a bright‐field image and its zoom‐in of a tiny 10 μm elephant printed inside a living cell. 3D confocal imaging was performed to confirm that the structure does indeed lie inside the cell (Figure 1e). Additionally, two or more separate structures can be printed inside a single cell (Figure S3b,c). The printing of a 10 μm structure takes 3–10 s, depending on the structure volume. Due to the high viscosity of the photoresist, small droplet size, and very short printing times, the printed structure movement during printing is negligible. Therefore, there is no need for additional supporting elements as in a typical 3D printing.

## 3 Viability and Cellular Behavior of Cells With Printed Structures

**FIGURE 2:**
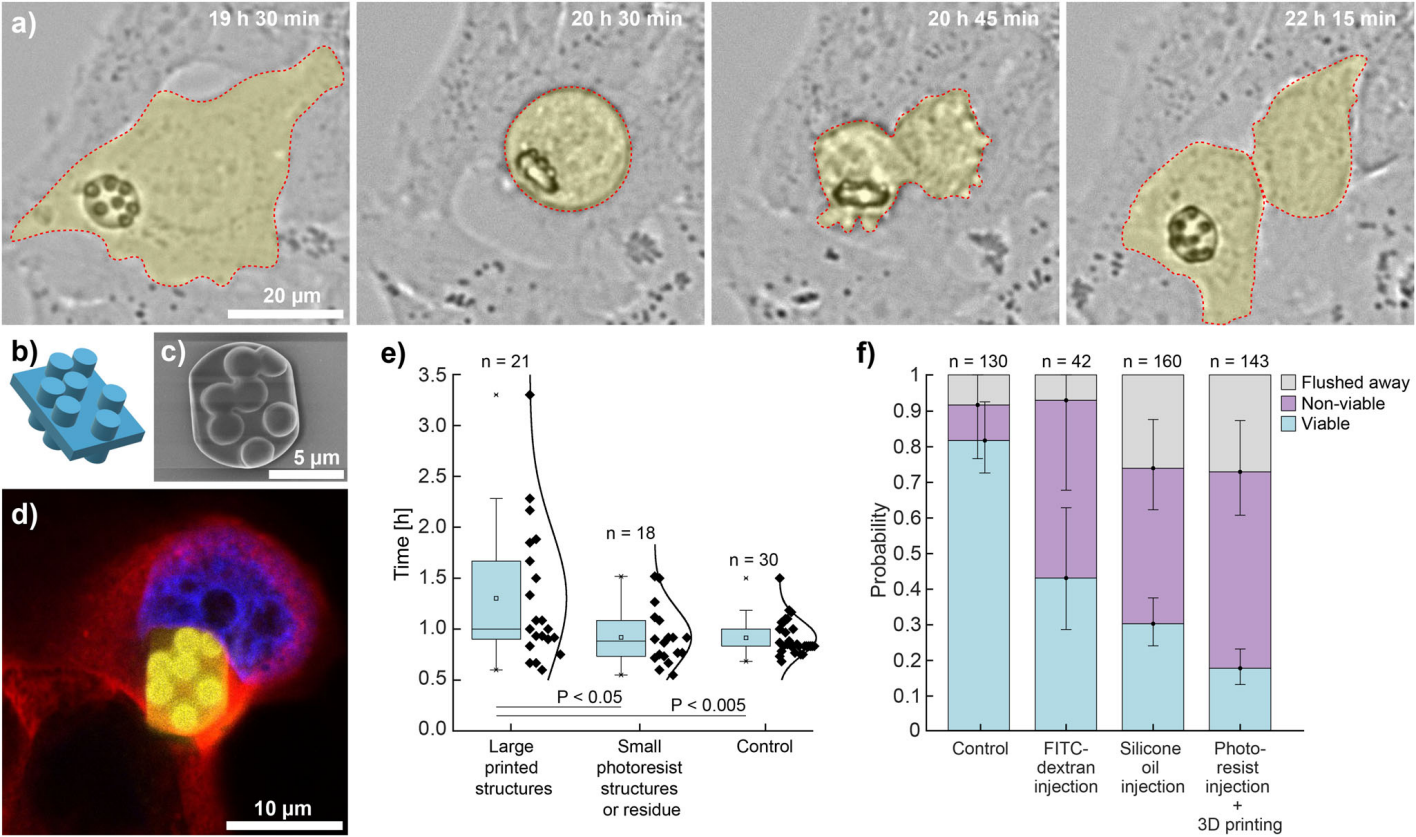
Viability of cells with printed structures. (a) A HeLa cell containing a printed structure undergoes cell division, starting in the second panel, 20.5 h after injection. (b) A CAD design of the structure ‐ J. Stefan Institute logo. (c) SEM image of the same structure when printed in a droplet of IP‐S photoresist. The structure is truncated at the edges since it was slightly larger than the droplet. (d) Confocal image of a HeLa cell, containing the polymerized structure. The cell nucleus visibly deforms and gives way to the structure. (e) Distributions of cell‐division times for cells with large printed structures, for cells with small photoresist printed structures or residue and for non‐treated cells. For statistical significance, a two‐sample t‐test has been used. (f) In the viability study, HeLa cells were monitored over the course of 24 h. We show results for cells containing polymerized IP‐S structures and three controls: first, cells that were not manipulated in any way, second, cells in which the membrane was penetrated and only a small amount of FITC‐dextran‐dyed medium was injected, and third, cells, where injection of a droplet of inert silicone oil, corresponding in size to the photoresist droplets, caused a sudden increase in the cell volume. In all control experiments, the cells were exposed to the same environmental conditions as the cells in which the structures were printed. Error bars represent standard error.

One of the crucial parts of this study was determining the effect of 3D printing on the cells, specifically the cell viability. Already by time‐lapse imaging, it can be observed that viable cells containing the printed structures have normal morphology (Video S1). The cells were observed to divide, and the structure was passed to one of the daughter cells (Figure 2a; Video S2). The printed structure shown in Figure 2a was in the shape of the J. Stefan Institute logo. Its computer‐aided design (CAD) and scanning electron microscopy (SEM) image are shown in Figure 2b,c. Confocal images show that the internal structure of the cells, especially the nucleus, deforms to accommodate the printed structures (Figure 2d). This is further confirmed by fluorescence imaging of the actin filaments (Figure S4 and Video S3).

Large printed structures inside cells could affect cell biology. Indeed, the cells containing large printed structures have a longer division time compared to cells with small printed structures (<3 μm) or photoresist residue and control cells that were not injected with the photoresist (Figure 2e; Figure S5). In cells with the large printed structures, the time required for the cell division is extended by 1 h and up to a few hours. In comparison, cells with small printed structures or photoresist residue and control cells have shown significantly different cell‐division times. For statistical significance, a two‐sample t‐test has been used. There is also an indication that large 3D printed structures can cause increased cell mobility of the daughter cells after mitosis (see Supplementary Discussion and Video S4). With this, we demonstrated that we can create cells with different biological properties.

We further quantified how micropipette membrane penetration, sudden cellular volume increase, and membrane reshaping due to the droplet injection, toxicity of the photoresist, and laser illumination, affect the viability of cells (Figure 2f). We checked how the membrane penetration influences viability by injecting a small volume of a dyed medium, commonly used in microinjection experiments. To check how the sudden volume increase affects the viability, inert silicone oil was injected into the cells, forming a droplet comparable in size to typical photoresist droplets. The effects of photoresist toxicity and illumination were tested simultaneously.

Microinjection is a method standard for delivering foreign materials into individual cells. Membrane penetration is known to cause damage that sometimes leads to cell death. In our case, already in the control without injection, there were 10% non‐viable cells after 24 h, probably due to the prolonged handling of cells at ambient conditions. By comparing the FITC‐dextran injected cells to the control, we can see that the microinjection causes an increase of non‐viable cells to 50%. This percentage is also similar for the silicone‐oil‐injected cells (44%) and for cells with structures printed inside (55%). This suggests that membrane penetration, which is common in these three cases, is the primary cause of cell death. However, there may be other factors, such as mechanical stress from inserting a large object into the cell and potential toxicity from the photoresist or illumination. When comparing the number of cells flushed away during culture medium exchange, we see no link to membrane penetration, as the percentages of flushed cells are similar for control and FITC‐dextran‐injected cells. Conversely, flushing is more significant in cells injected with silicone oil and photoresist, indicating that injecting a droplet raises the chance of cells being flushed away. After injection, the membrane must reshape considerably to hold the droplet, likely weakening the cell's attachment to the substrate and making it easier for cells to detach during pipetting.

While our study was not specifically designed to optimize cell viability, the observed viability rates are comparable to those of various commonly used methods in cell biology. For instance, in the case of microinjection, which was employed in our study, reported viability rates in the literature can vary significantly. They range from as low as 5%–15% for manual microinjections into neurons [21] to as high as 96% for semi‐automated microrobotic microinjections into adherent cells [22]. These viability rates are also comparable to the ones for lipofection and electroporation, which are standard methods for transfecting cells. The transfection of CRISPR/Cas9 can result in viability rates below 50% [23, 24]. In the future, the overall viability of the cells could be improved by using bio‐compatible photoresists explicitly developed for printing inside cells, optimizing the injection procedure, as well as cell handling and printing at physiological conditions inside a cell incubator.

## 4 Quality of Printed Structures

In TPP lithography, the structures are usually printed in a bulk photoresist. In our case, the printing occurs in a droplet of photoresist within a cell, therefore experiencing a substantial refractive‐index mismatch. The refractive index of the unpolymerized photoresist is 1.48, and that of the cytoplasm is between 1.36 and 1.39. The refraction of the laser light through a curved droplet surface could result in distortions of the structures being printed and a decreased resolution. When viewed under an optical microscope, the structures printed in cells do not appear deformed. Only when the structure is larger than the droplet, the printed object can be truncated at the edge of the droplet (Figure 2a,c).

**FIGURE 3:**
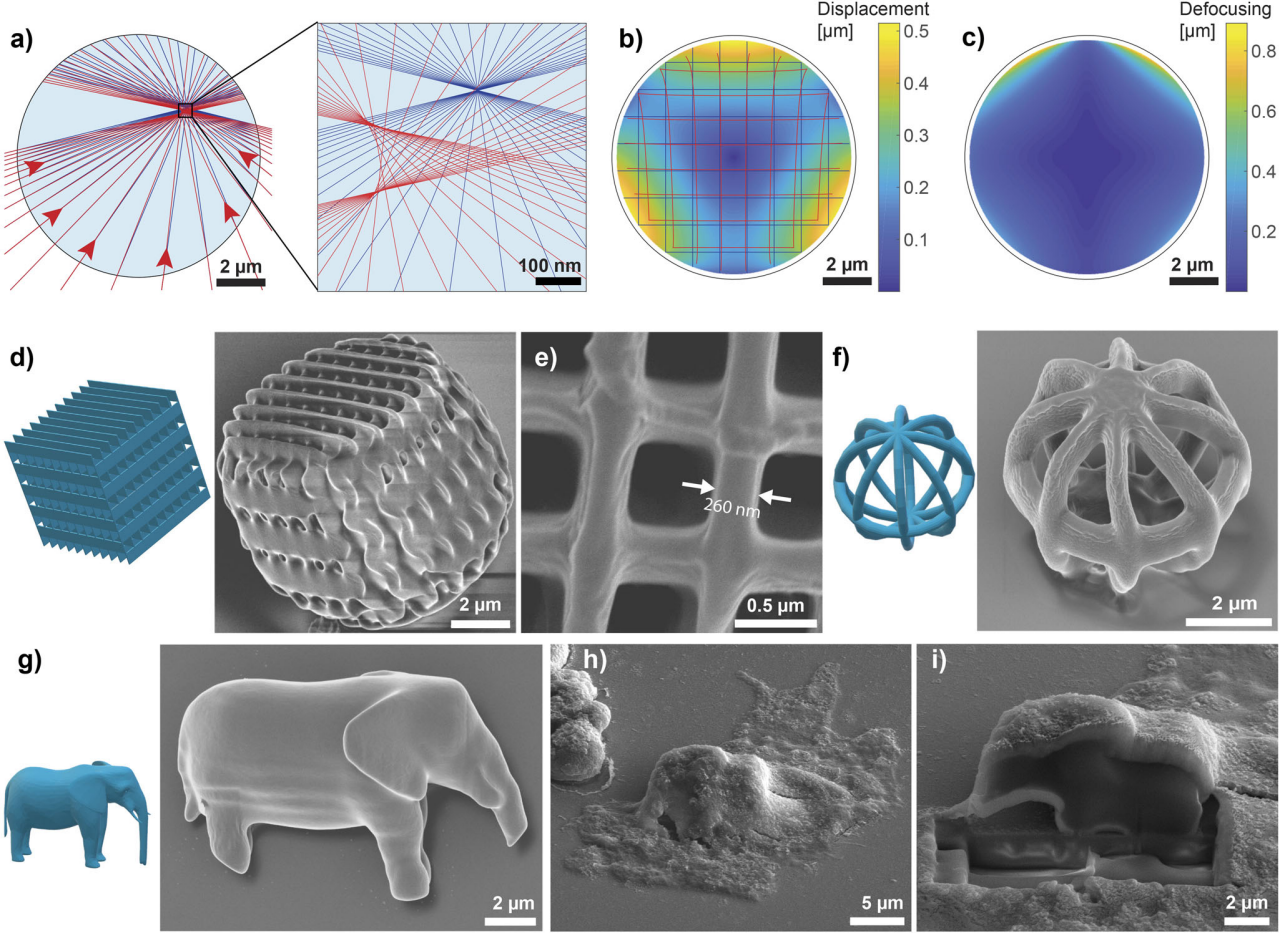
Printing quality analysis. (a) Focused beams of light with (red) or without (blue) the droplet present. The beams are drawn in one plane only. Due to the refraction on the droplet surface, the beams are not focused to a single point, and the overall focal point is shifted, as seen in the close‐up on the right. (b) A color plot, representing focal displacements in a meridian plane, as obtained in a 3D simulation, with a deformation of a square grid plotted for clarity. The image represents displacements in a selected plane intersecting the poles of the droplet; however, due to the rotational symmetry of the problem, the results are equal for all meridian planes. (c) A color plot, representing the focal spot size in a meridian plane as obtained in a 3D simulation. (d) A CAD design of a woodpile structure and a SEM image of the corresponding structure printed in a photoresist droplet. (e) A top‐view SEM image of a grid in a structure, made by the same CAD design. (f) A CAD design and a SEM image of a 6 μm hollow spherical structure printed in a droplet. (g) A CAD design and an SEM image of an elephant printed in a droplet (model downloaded from Tinkercad Shapes Library, designed by the user “geometricity”). (h) A SEM image of a cell with an elephant‐shaped structure printed inside it. (i) A cross‐section of the cell and the elephant‐shaped structure as obtained by focused ion beam milling.

We performed a ray‐optics simulation, where we analyzed how printing inside a droplet with a different refractive index than the surrounding medium affects the shape of the printed structures. Due to refraction at the droplet surface, the focus is shifted, and the focal spot size increased (Figure 3a). This image displays the difference between the non‐refracted beams (blue) and the beams refracted at the droplet surface (red) when focusing to a point within the droplet. For better visualization, the beams are drawn for a 2D case, whereas all the quantitative results were calculated in 3 dimensions. We have found that the displacements of the focus are small and amount to a maximum of 0.5 μm near the droplet edge (Figure 3b). The deformation is already very small, but if needed, it could be easily completely eliminated by pre‐deforming the model in the opposite direction to the expected shifts in the droplet due to diffraction.

The defocusing, shown in a meridian plane (Figure 3c), is below the diffraction limit of 400 nm in more than 90% of the droplet volume. This means that the structures can be printed inside droplets with a resolution very close to the one achievable in bulk. Additional results from the simulation are presented in Figure S6.

To experimentally confirm that there are no significant distortions or loss of resolution of the structures printed within droplets, we took SEM images of the structures printed in 10‐15 μm droplets. In this case, the droplets were not in cells but in a mixture of glycerol and water, which had a refractive index close to that of the cytoplasm. We printed a woodpile structure, built from walls of single‐voxel width, to evaluate the achievable printing resolution (Figure 3d). The design was slightly larger than the droplet. Therefore, the printed structure is slightly truncated on the sides. However, the grid on top appears flat and does not contain visible deformations, confirming that the in‐droplet printing yields structures of high fidelity. The top view of the grid of another structure printed by the same design (Figure 3e) further confirms a good printing quality, with the grid exhibiting walls as thin as 260 nm with a periodicity of ∼ 0.8 μm. In this study, the printing parameters were not specifically optimized to achieve the highest resolution. However, the achieved 260‐nm feature size is not far from the one achievable in bulk for the same photoresist. SEM images of other designs, printed in droplets, are shown in Figure 3f,g, and Figure S7. This demonstrates that any 3D structure can be printed with high resolution and fidelity, including structures with voids (Figure 3f). Due to the limited resolution of the 3D printing, some very small features appear thicker compared to the original design (e.g., elephant tail and tusks) or blurred‐out (elephant eyes).

The structures printed in cells are of comparable quality to the ones printed in the photoresist droplets in water. The resolution of the prints is similar in both cases, as can be seen by comparing the single‐voxel walls of the woodpile structures in Figure 3e and Figure S8a,b. Cross‐sections of an elephant‐shaped structure that were obtained by focused ion beam (FIB) milling are homogeneous inside and do not exhibit porosity on the 100‐nm scale (Figure 3i; Figure S8c). The Young's modulus of IP‐S is 2.1 GPa, which is many orders of magnitude more than that of the cells. This means the structure is completely stiff compared to the cell. This can also be seen in the experiments, where even thin printed structures do not deform during cell migration and division (Video S5).

## 5 Applications of the Printed Structures

**FIGURE 4:**
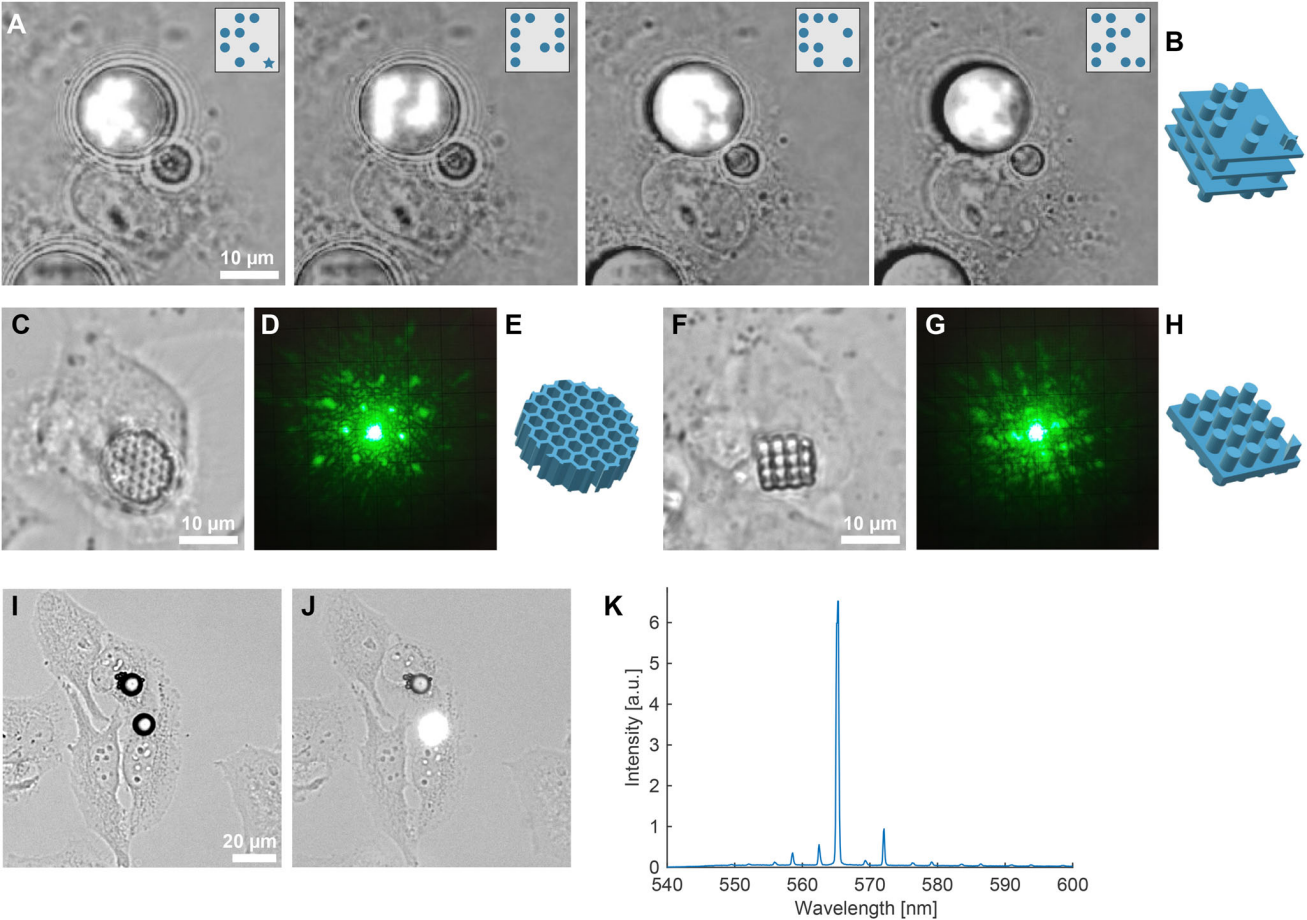
Applications. (a) Layer‐by‐layer printing of a 3D barcode inside a photoresist droplet within a HeLa cell. Insets show designs of the corresponding layer. (b) CAD design of the 3D barcode. (c) A hexagonal diffraction grating printed inside a HeLa cell, (d) its diffraction pattern, and (e) the CAD design. (f) A square diffraction grating printed inside a HeLa cell, (g) its diffraction pattern, and (h) the CAD design. (i) Two WGM lasers made by single‐photon polymerization of a droplet of high refractive index photoresist IP‐n162, each injected into its separate HeLa cell. (j) Laser emission from a WGM laser within a cell, and (k) the corresponding emission spectrum.

There are many promising applications of printing within living cells. One of the applications we explored is barcoding, which involves writing a specific code to each cell for identification and long‐term tracking of the cell. We have designed a 3D graphical barcode composed of 4 stacked 4 × 4 grids of cylinders (Figure 4a,b; Figure S7c and Video S6). Each of the 64 spaces can be occupied or not. In the middle two layers, at least one cylinder should be present so that the structure does not fall apart. One cylinder on the top corner is also always present and is star‐shaped so that the orientation of the structure can be determined. Therefore, we can encode 61 bits of information, giving a total of ($2\cdot 10^{18}$) unique barcodes. This number far exceeds the number of cells in the human body. For real‐life applications, much smaller barcodes could be used. Compared to most other cell barcoding techniques [19, 25], our method uniquely allows for encoding predefined information into each cell, rather than relying on random barcodes. However, it is limited to barcoding a small number of cells and is presented here primarily as a proof of concept.

For remote reading, diffraction gratings of different designs can also be used as cell barcodes (Figure 4c–h). By illuminating them with a laser, a diffraction pattern that depends on the symmetry and periodicity of the structure can be read from a distance. Additionally, as the diffraction pattern is orientation‐dependent, a diffraction grating could also be used to remotely measure cellular rotations in all three axes.

We have further explored whether we can manufacture an active optical device, specifically a microlaser, inside a cell [18, 26]. A microsphere with a refractive index higher than the surrounding medium and containing a fluorescent dye can act as a whispering gallery mode (WGM) laser. By injecting fluorescently doped high‐refractive‐index photoresist IP‐n162 into live cells and polymerizing the whole droplets, we managed to produce WGM lasers with diameters as small as 9 μm (Figure 4i–k; Figure S9). While WGM lasers, uptaken into the cells through phagocytosis, have been used for cell tagging and intracellular sensing before [15, 19, 26], our approach enables their use in cells that cannot uptake larger particles. The high‐refractive‐index photoresist that we used appears more cytotoxic than IP‐S, causing all of the injected cells to either die (80%) during the course of 22 h, or at least be visibly affected. However, with the development of new bio‐compatible photo‐curable materials, we expect this setback can be overcome.

Instead of using photoresists only slightly soluble in water, it would also be possible to use a water‐soluble photoresist that would mix completely with the cytosol, enabling printing anywhere within the cell. This would enable the isolation of a specific part of the cell within a newly formed compartment, for example, to study the function of individual organelles or to investigate signaling pathways by physically blocking specific pathways. Instead of inactivating or physically destroying organelles to study their regeneration or biogenesis, as other groups have done [27, 28], our method would enable fixing them through photo‐polymerization. By selectively polymerizing whole cells, this approach would facilitate the study of how mechanical properties influence various cellular behaviors and model diseases associated with changes in tissue stiffness. One way of delivering the water‐soluble photoresist into cells would be to use a membrane‐permeable photoresist [11, 29]. The other method, which was used here, is to inject a water‐soluble hydrogel‐based photoresist into the cells by using a micropipette. Preliminary results of this approach are presented in the Supplementary Discussion and Figure S10.

## 6 Conclusion

For the first time, we printed various polymeric structures directly inside living cells. While microstructures can be introduced into cells by phagocytosis, as shown before [16, 30], only a small number of cell types have phagocytotic ability. Also, in this case, the structure ends up in a phagosome and is not directly inside the cytosol. Here, we demonstrate a universal method for delivering larger objects into cells.

There are many possible applications of structures printed inside the cells, well beyond what has been shown here. Especially interesting is the prospect of printing functional structures, which would change the properties of cells beyond what has been possible till now by conventional bioengineering tools. Structures, such as micro‐levers, springs, cages, and barriers printed in cells, could be used to apply controlled mechanical forces to intracellular components, introduce chirality, and modify cell shape and cellular mechanics. Such modified cells could help us study cell mechanics, cellular division, mechano‐transduction, and mechanically induced differentiation of cells in culture and in tissues. We have already observed different behavior of cells with printed structures, opening the possibility of controllably changing cell phenotype.

Here, we printed diffraction gratings and lasers, but other optical devices, such as lenses [31] and waveguides for controlled light delivery and imaging, could also be used.

With the fast development of new functional materials for photolithography, TPP could be used to fabricate various active devices inside living cells. Functional micro‐devices are produced by 4D TPP printing [32, 33] using different functional materials [34], responsive to various external stimuli [35, 36], such as light [37, 38], temperature [39], pH [40], sugar concentration [41], and magnetic field [42]. This would enable both novel sensing modalities and the creation of active components [43] and even microrobots inside cells. Further, TPP can also be performed with conductive materials [44, 45], even inside living organisms [46]. Conductive micro‐structures printed in live cells could lead to new intracellular electrophysiology modalities [47, 48]. By using nanodiamond‐embedded photoresists, even quantum sensing [49] might be possible inside cells. Further, printing structures loaded with drugs [50] with controlled size, shape, and position within the cell could enable spatio‐temporally controlled intracellular drug release.

Intracellular 3D printing offers an unprecedented degree of control over the cellular interior, allowing the integration of synthetic structures with native biological functions. This platform could allow for reconfiguration of cellular architecture, embed logic or mechanical components within the cytoplasm, and design cells with enhanced or entirely new properties. These developments could have far‐reaching implications for synthetic biology, intracellular sensing, and the fundamental study of cellular structure and function.

## 7 Experimental Section

### 7.1 Materials for Injection

Materials used for injection into live cells: IP‐S photoresist (Nanoscribe GmbH) with a refractive index 1.486 in liquid form and 1.515 in polymerized form, IP‐n162 photoresist (Nanoscribe GmbH) with a refractive index of 1.604 in liquid form and 1.622 in polymerized form, dyed with Pyrromethene 597 (Exciton, USA), and Tris‐EDTA (TE) buffer dyed with 1 mM FITC‐dextran (10 kDa, Sigma–Aldrich). For silicone oil injection, two inert silicon oils were used yielding similar results, namely Antievaporation oil (Ibidi, Germany) and Poly(dimethylsiloxane‐co‐methylphenylsiloxane) (Dow Corning, USA). Other photoresists tested for cellular injection and printing: IP‐Visio, IP‐L, IP‐Dip, and IP‐n162 (all by Nanoscribe GmbH).

### 7.2 Cell Culture

HeLa cells (ATCC, CCL‐2) were seeded into a well, consisting of a silicone layer with a 10 mm circular cut‐out (Silicone Isolators, Grace Bio‐labs) on a previously cleaned coverslip, 1–2 days before the printing. Cells were grown overnight in the cell‐culture medium DMEM (Dulbecco's Modified Eagle Medium, DMEM (1X) + GlutaMAX, Gibco) supplemented with 10% fetal bovine serum and 1% penicillin–streptomycin in an incubator (37 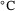, 5% ${\mathrm{CO}}_{2}$) to achieve approximately 70% confluence. Before cell injection, the cell growth medium was exchanged for LCIS (Live Cell Imaging Solution, Molecular Probes, Invitrogen) supplemented with 10 mM glucose. The LCIS was used because the injection and printing took place at room conditions.

### 7.3 Micropipette Preparation

The micropipettes were pulled on the P1000 Micropipette Puller (Sutter Instruments) from borosilicate glass capillaries with a filament (outer diameter 1 mm, inner diameter 0.78 mm, BF100‐78‐10, Sutter Instruments) using the following program settings: heat 467, pull 90, velocity 70, delay 90, pressure 220, resulting in a micropipette tip with a sub‐micron outer diameter (Figure S1a). On the day of the injections, the previously pulled micropipettes were silanized to improve wetting and facilitate resin flow. For 3 min, the inside of the micropipette was exposed to a vapour of silane (Dimethyloctadecyl[3‐(trimethoxysilyl)propyl]ammonium chloride solution, Aldrich, Germany), produced by heating the solution to 60 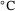. Afterward, the silane was baked for 20 min at 120 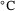. After silanization, the micropipette was filled with the photoresist. For injection of the TE buffer dyed with FITC‐dextran, the silanization step was not performed.

### 7.4 Injection Into Cells

Injection of photoresists and other materials into the cells was performed with a FemtoJet 4i microinjector (Eppendorf), coupled with an InjectMan 4 micromanipulator (Eppendorf). For injection, we randomly selected cells from the ones that appeared well attached to the substrate (well spread‐out). Otherwise, they were very likely to detach during injection and stick to the micropipette. Microinjection was performed at room temperature, without the added ${\mathrm{CO}}_{2}$, in LCIS. During one experiment, up to 40 droplets were injected. Typical injection pressure $p_{i}$ and injection time $t_{i}$ for IP‐S photoresist were $p_{i}=5000\;\text{hPa}$, $t_{i}=25\;s$, for silicon oil $p_{i}=4000\;\text{hPa}$, $t_{i}=1.7\;s$, for TE buffer dyed with FITC‐dextran $p_{i}=1000\;\text{hPa}$, $t_{i}=4\;s$, and for IP‐n162 photoresist $p_{i}=5000\;\text{hPa}$, $t_{i}=40-90\;s$.

### 7.5 3D‐Printing Protocol

After taking the sample out of the incubator, DMEM was exchanged for LCIS. This was followed by the microinjection step, which took approximately 30 min, and then the printing procedure commenced. A commercial system, Photonic Professional GT2 (Nanoscribe, Germany), was used for printing. The printing step lasted for approximately 45 min, and was performed at ambient conditions. Most of this time was used to mount (and later dismount) the sample and to manually search and appropriately position each droplet into the location of the laser spot. After positioning, each droplet was illuminated with a 780 nm femtosecond laser through a high numerical aperture 63× objective (Zeiss ‐ 1.4 NA Oil DIC, Plan Polychromat). The system operated in the galvo mode, with the scan speed of 10 000 μm $s^{-1}$. Illumination of a single structure took 3–10 s, depending on the structure volume. After printing all the structures, the LCIS medium was exchanged for DMEM, and the sample was put into an incubator. The typical experimental timeline is shown in Figure S2.

The codes for printing different 3D structures were prepared in the proprietary Describe software, where the CAD design was automatically sliced into 100 nm layers, which would be illuminated one after the other. Within each plane, the laser was programmed to fill the structure by moving in parallel lines, separated by 100 nm. The structures were coded to be printed top‐down, so first the photoresist furthest away from the glass was to be illuminated and then layers closer and closer to the glass. Due to the high viscosity of the photoresist and short printing times, the subsidence of structures during printing was not problematic.

### 7.6 Analysis of Cell‐Division Times

We have quantified the time of cell division for three groups of cells. The first group comprises cell‐division times of cells, containing large (>5 μm) printed structures (n=21). The second group contains the division times of cells, where only a small (<3 μm) polymer structure can be observed. This structure can either be a residue of an unpolymerized photoresist droplet, or a very small polymerized structure obtained by illuminating only a small photoresist volume. The third group (control) comprises of cell‐division times of cells, that were not injected with the photoresist. The data were combined from several separate experiments to obtain enough data points.

The time of cell division was determined as the time between two individual stages of the mitosis cycle that can be most precisely determined from visual observations: first, the cell detachment from the surface and second, the late anaphase transition into the telophase stage after chromosome segregation (Figure S5). We measured the time interval between the two stages with approximately 5‐min temporal resolution, which was much smaller than the spread of the data.

### 7.7 Viability Study

To check the extent to which the microinjection damages the cells, 2 different sets of control experiments were performed. In one, a small amount of microinjection buffer (Tris‐EDTA (TE) buffer, typically used in DNA microinjection experiments) dyed with FITC‐dextran was injected into the cells to see how damaging the puncturing through the membrane was by itself. The dye was added to find the microinjected cells the next day, after the incubation. In another control experiment, inert silicone oil was injected into the cells. This was physically much more similar to the injection of the photoresist, as the silicone oil formed similarly sized droplets within the cells. Viability was assessed by performing a live/dead assay 24 h after the injection. We compared these results to the viability of the cells that were subjected to the same environmental conditions but were not mechanically manipulated in any way. Microinjection and printing were performed at room conditions, with cells submerged in a medium designed for room‐condition imaging. The timeline of changes of the outside conditions (temperature, medium exchanges, and the sample put into the incubator) was kept the same in all experiments. The first 75 min, the samples were kept at room conditions in the LCIS, then, the LCIS was exchanged for the cell‐growth medium DMEM, containing 20 nM Sytox Green Nucleic Acid Stain (Invitrogen) or 50 nM Sytox Deep Red Nucleic Acid Stain (Invitrogen). The Sytox stains were used as live/dead indicator dyes, labeling the nuclei of dead cells while being unable to cross the intact membranes of viable cells. The samples were imaged and put into the incubator. At 24 h, the samples were checked again to see how many of the observed cells were still viable. The Sytox staining was validated by simultaneous time‐lapse bright‐field imaging of the sample overnight, where a cell was determined to be non‐viable if it showed morphological signs of different stages of apoptosis (cell shrinkage, membrane blebbing and formation of apoptotic bodies). Cells identified as non‐viable by time‐lapse bright‐field imaging overlapped exactly with those exhibiting Sytox fluorescence, confirming the high reliability of Sytox staining. The bar chart in Figure 2f shows combined results obtained from multiple repetitions of the experiment. For Control, the data were combined from three independent experimental repetitions, for FITC‐dextran injection from two repetitions, for silicone‐oil injection from four repetitions and for IPS‐photoresist injection and 3D printing from five independent repetitions.

### 7.8 Conventional and Confocal Microscopy

Brightfield imaging was performed on an inverted microscope (Nikon Ti2). The samples were placed into a tabletop incubator. To reduce the photo‐toxicity, the white LED illumination was set to a low value and filtered with a 600 nm longpass filter. Imaging was performed with 10×, 20×, and 40× objectives with a monochrome camera (Ximea xiC MC203MG‐SY‐UB).

For viability studies, a bright‐field image was taken after the photoresist injection, after printing and medium exchange, and at the end of the 24‐h experiment. After medium exchange and at the end of the experiment, a fluorescence image, where the nuclei of all the nonviable cells were labelled green or red, depending on the Sytox dye used, was taken in addition to the bright‐field image. In some experimental repetitions, time‐lapse imaging was performed by taking a bright‐field image every 10 min (or every 5 min) during the 24‐h experiment. Additionally, every few hours and at the end of the experiment, a fluorescence image was taken. Counting of viable and nonviable cells was done manually.

For the time‐lapse imaging of the cells in the fluorescence mode presented in Video S1, the cell nuclei were labeled with NucSpot Live 488 (by Biotium, USA) and the cell membranes with CellMask Orange dye (by Invitrogen, USA). A time‐lapse image sequence was recorded by a color camera (IDS, UI‐3280CP‐C‐HQ R2).

For the time‐lapse imaging of the cells in the fluorescence mode presented in Video S3 and Figure S4, the cell nuclei were labeled with NucSpot Live 488 (Biotium, USA) and the actin filaments with SPY555‐actin dye (Spirochrome). A time‐lapse image sequence was recorded by a monochrome camera (Ximea xiC MC203MG‐SY‐UB), by separately imaging in green and red channels. The grayscale images from separate channels were artificially colored and combined.

For confocal imaging, the samples were fixed by immersion in 1% paraformaldehyde (PFA, Biotium, USA) for 5 min, and a subsequent immersion in 2% PFA for 10 min, washed with phosphate‐buffered saline (PBS, Gibco) and labelled with CellMask Deep Red (Invitrogen, USA) for 10 min and NucSpot Live 488 (Biotium, USA) for 30 min at room temperature. The IP‐S structures did not need to be additionally labeled, as the photoresist was fluorescent by itself. Confocal imaging was performed on a customized confocal microscope (Abberior). The grayscale images from separate channels were artificially colored and combined. Brightness and contrast were adjusted separately for each channel.

### 7.9 Scanning Electron Microscopy (SEM)

SEM imaging was performed on two different types of samples. The first type was structures printed in IP‐S photoresist droplets dispersed in a glycerol solution in deionized water (1:3 volume ratio) in a 50 μl well. This solution has a refractive index of 1.365. After printing, the well was submerged in 5 mL deionized water for a couple of hours for the unpolymerized photoresist to dissolve. Then, the structures were dried. The second type of samples were structures printed in cells. After printing and allowing a two‐hour period for the non‐polymerized photoresist to dissolve, the cells were prepared for high‐vacuum FIB‐SEM following a protocol adapted from a recent study [51]. To minimize shrinkage and collapse associated with excessive chemical fixation, cells were exposed to 2.5% glutaraldehyde (GA) for only 60 s. This short fixation step enabled improved preservation of structural integrity and natural morphology. Subsequently, samples were washed with PBS and 150 mM ammonium acetate to remove buffer‐derived salt crystals. After gentle blotting with lint‐free paper to eliminate excess solution, samples were drop‐cast with liquid propane for rapid cryo‐fixation, thereby preventing crystalline ice formation, and transferred to a freeze‐dryer (Kambič, Lio‐5PLT) for 2 days. After drying, both types of samples were coated with an approximately 10 nm Au/Pd layer using a Gatan 682 Precision Etching and Coating System (PECS). SEM imaging was performed using a FEI Helios Nanolab 650 in immersion mode for high‐resolution surface imaging (2 keV electron acceleration voltage, 25 pA beam current, $10^{-6}$ hPa chamber vacuum) and in field free mode for larger field of view imaging (10 keV, 25 pA). To enhance 3D visualization and enable FIB milling of the 3D printed objects, the stage was tilted up to 52 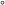. For FIB milling of cells to obtain cross‐sectional images of the printed structures, a gallium ion source was employed (beam energy 5–30 keV; beam current 0.5–2.5 nA).

### 7.10 Microlaser Operation and Analysis

A WGM microlaser consisted of a high‐refractive‐index photoresist dyed with a fluorescent dye (Pyrromethene 597) to act as a gain medium. After injection of the photoresist droplet into the cell, the whole droplet was shortly (5 s) illuminated with a UV LED light to polymerize (one‐photon process). Then, the droplet was pumped with a tunable pulsed nanosecond laser (NT242, Ekspla, Lithuania) with a wavelength set to 532 nm and a repetition rate of 10 Hz. The emission spectrum was measured with a spectrometer (Andor Shamrock, SR‐500i‐D1) equipped with a CCD camera (Newton, DU940P‐BV), with a spectral resolution of 0.13 nm. To characterize the laser, a model system was used, with a photoresist/dye droplet immersed in a water environment instead of in a cell. A typical two‐slope curve was obtained when increasing the pump laser pulse energy and measuring the intensity of one of the spectral peaks, indicating a lasing threshold of approximately 0.07 nJ $\mu m^{-2}$ (Figure S9c). A decrease of the linewidth was also observed at this value.

### 7.11 Simulations

The precision of the printing process in terms of displacement and width of the beam at different positions inside the droplet was calculated by simulating the propagation of rays. A sufficient number (∼2500, see Figure S6b) of rays with their origins and angles of propagation were selected. The origins were homogeneously distributed in a plane below the droplet, while the angles were such that all the rays would converge to the same focal position in the absence of refraction. The maximum entrance angle was calculated based on the numerical aperture of the objective and the refractive indices of the materials used in the experiment. Due to refraction at the curved surface of the droplet, different rays refract differently, leading to a wider and displaced area of ray intersections. The new focal position was assigned by looking at the distribution of rays in each vertical plane. The plane where the variance of ray positions was the smallest was defined as the z position of the focus, while x and y positions were taken as the center of mass in that plane. Displacement was then calculated as the difference between this position and the position if no refraction would take place. The focus width or resolution was defined as the variance of ray positions in the newly‐assigned focal plane. In the simulation, the ratio between the refractive indices of unpolymerized photoresist IP‐S and cytoplasm was 1.08. The droplet diameter was 10 μm. For the calculation of the focus displacement and defocusing, the simulation was ran only for the volume within the 0.96 of the droplet radius (Figure 3b,c) as at the upper edges of the droplet the rays were refracted in a manner that they can become parallel and do not intersect at all, resulting in the divergence of the simulation. For calculating the percentage of volume, where defocusing was below the diffraction limit, the simulation additionally included radii up to the full droplet radius, and the diverging points were attributed a defocusing value above the diffraction limit.

### 7.12 Statistical Analysis

Statistical analysis was performed for the results presented in Figure 2e,f. The data was not pre‐processed, and no data points were discarded. The sample size n for each group was given in the figure. The data in Figure 2e is presented with box plots, with each box covering values between the 25th and 75th percentiles. Next to the box plots, individual data points with the fitted distributions were shown. The results were combined from 7 independent experimental repetitions. To assess statistical significance, a two‐sample t‐test was used with the P‐values provided in the figure. Statistical analysis was performed using OriginPro 2018 (OriginLab Corporation, Northampton, MA, USA). In Figure 2f, the data are presented with bar charts, with error bars representing standard error. The results were combined from multiple independent experimental repetitions, as described in the *Experimental section, Viability study*.

## Author Contributions

M. M. conducted experiments and analyzed the results. A. K. performed simulations and analyzed the results. In initial experiments, U. J. performed the 3D‐printing part of the experiment and later provided valuable insight regarding the 3D printing. R. P. analyzed the results, prepared the samples for SEM/FIB imaging and took SEM images. M.H. conceived the original idea, designed and supervised the study. M. M. and M. H. wrote the manuscript with input from the other authors. All authors approved the final version of the paper.

## Conflicts of Interest

The authors declare no conflicts of interest.

## Supporting information


**Supporting File**: adma71870‐sup‐0001‐SuppMat.pdf

**Supporting File1**: adma71870‐sup‐0003‐VideoS2.mp4

**Supporting File2**: adma71870‐sup‐0003‐VideoS2.mp4

**Supporting File3**: adma71870‐sup‐0004‐VideoS3.mp4

**Supporting File4**: adma71870‐sup‐0005‐VideoS4.mp4

**Supporting File5**: adma71870‐sup‐0006‐VideoS5.mp4

**Supporting File6**: adma71870‐sup‐0007‐VideoS6.mp4

## Data Availability

The data that support the findings of this study are openly available in [Zenodo] at [https://doi.org/10.5281/zenodo.16743381], reference number [16743381].
