# Supplementary material for: Two‐Photon 3D Printing of Functional Microstructures Inside Living Cells
Source: Adv Mater. 2026 Jan 14;38(25):e19286. doi: 10.1002/adma.202519286 (PMC13137771; doi:10.1002/adma.202519286)
Supplement: Supplementary file 1 — Supporting File: adma71870‐sup‐0001‐SuppMat.pdf [file ADMA-38-e19286-s003.pdf]

Supplementary Information - Two-photon 3D printing of functional microstructures inside living cells

Maruša Mur[1], Aljaž Kavčič[1,2], Uroš Jagodič[1], Rok Podlipec[1], and Matjaž Humar*[1,2,3]

[1]Department of Condensed Matter Physics, J. Stefan Institute, Jamova 39, SI-1000 Ljubljana, Slovenia
[2]Faculty of Mathematics and Physics, University of Ljubljana, Jadranska 19, SI-1000 Ljubljana, Slovenia
[3]CENN Nanocenter, Jamova 39, SI-1000 Ljubljana, Slovenia
*E-mail: matjaz.humar@ijs.si

Supplementary Figures

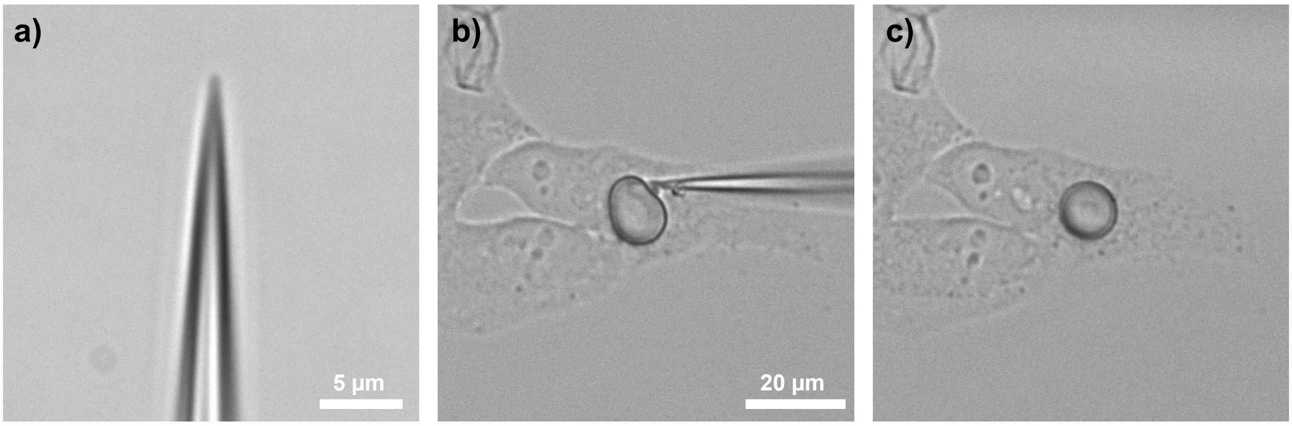

Supplementary Figure 1: *Microinjection.* ***(a)*** *Microinjection tip used for intracellular injections.* ***(b)*** *Injecting into a HeLa cell.* ***(c)*** *The same cell after injection.*

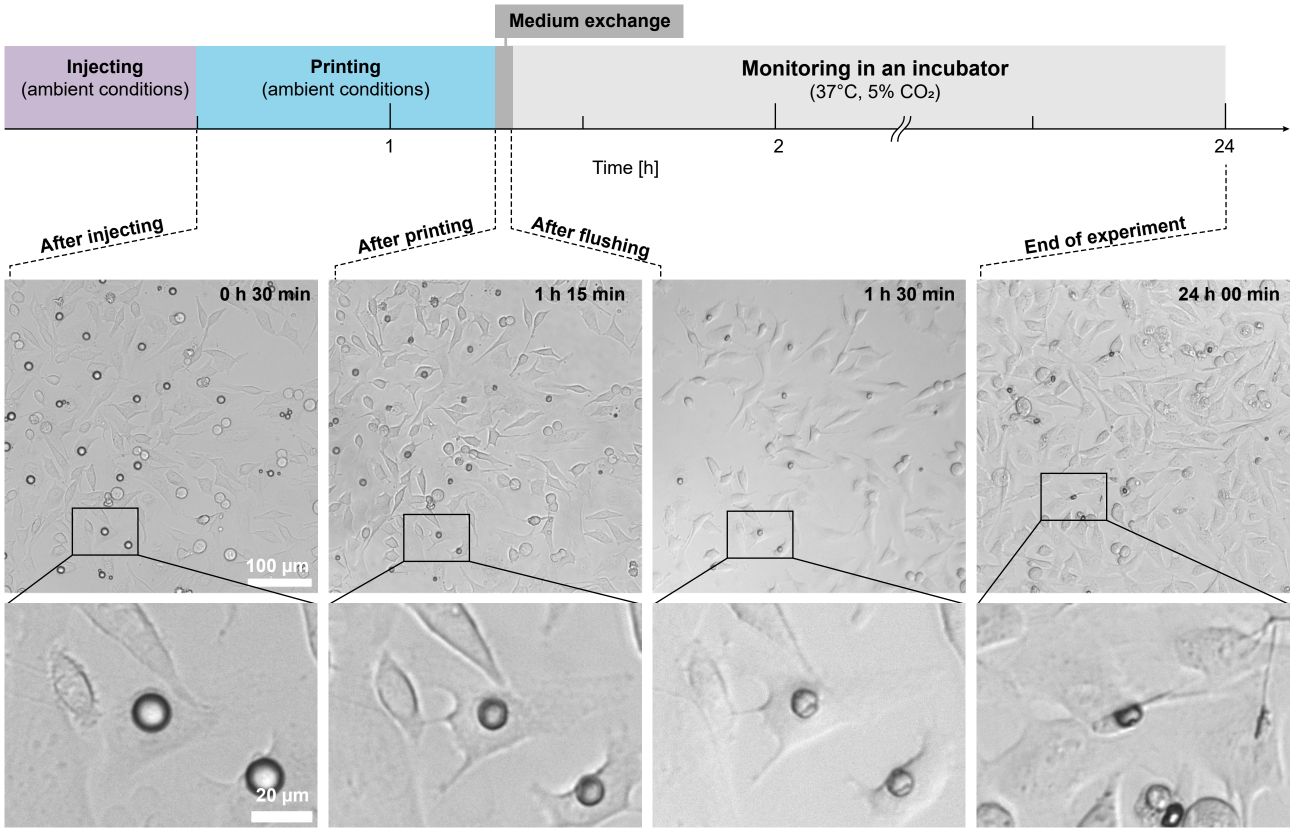

Supplementary Figure 2: *Typical experimental timeline. After taking the sample from the incubator, the cell culture medium is exchanged for live imaging solution and photoresist droplets get injected into the cells. The injections (typically 40 per sample) take place at ambient conditions and last approximately 30 min (all combined). Afterwards, the sample is inserted into the commercial system for two-photon photo-polymerization. Photoresist droplets in the cells are manually positioned and illuminated with a femtosecond laser following a pre-programmed path. While illuminating one structure takes only up to 20 s, it can take up to 45 min to position and illuminate all droplets in a sample. After the sample is taken out of the machine for TPP, the medium is again exchanged for the typical culture medium and the sample is put into the table-top incubator (37 °C, 5 % CO_2) where it can be imaged and monitored for a long time.*

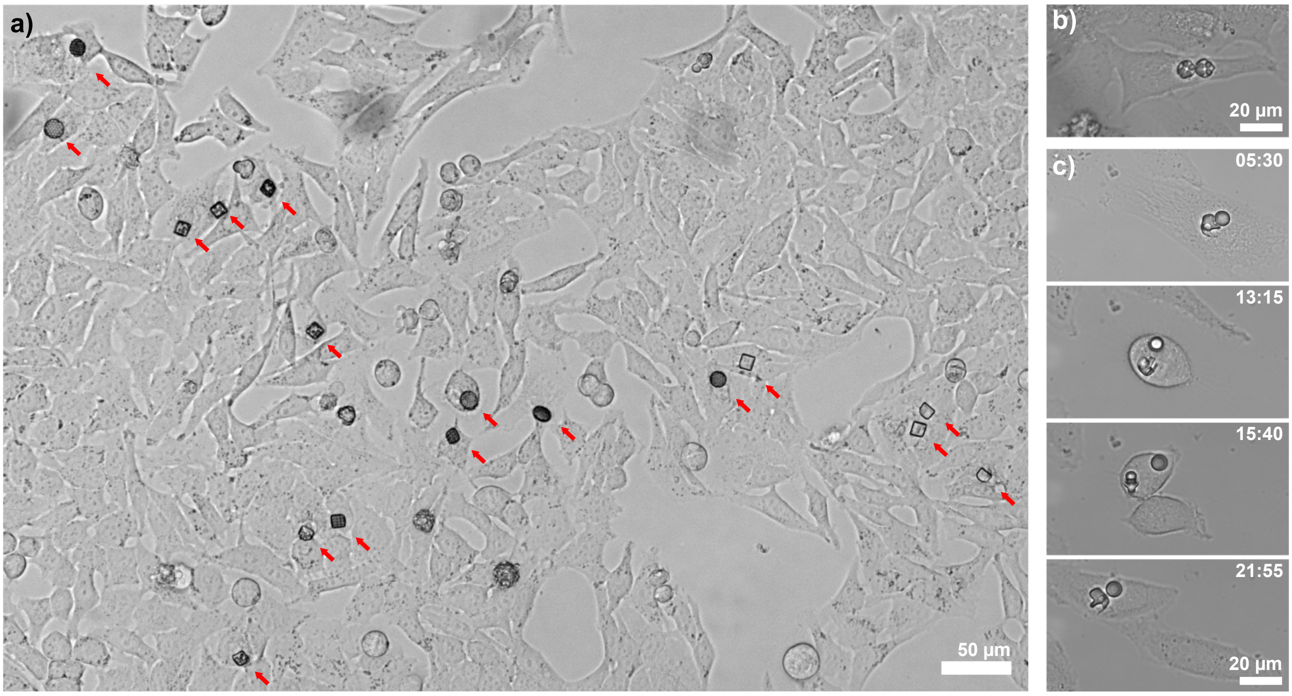

Supplementary Figure 3: *Numerous structures printed inside HeLa cells.* ***(a)*** *Several printed structures within one sample.* ***(b)*** *A cell containing two structures, printed inside. Two separate photoresist droplets had to be injected into the cell to achieve this.* ***(c)*** *A time-sequence of images showing a dividing cell that contains two structures - an elephant and a ball. Both structures end up in the same daughter cell. The full time-lapse is shown in Supplementary Video 5.*

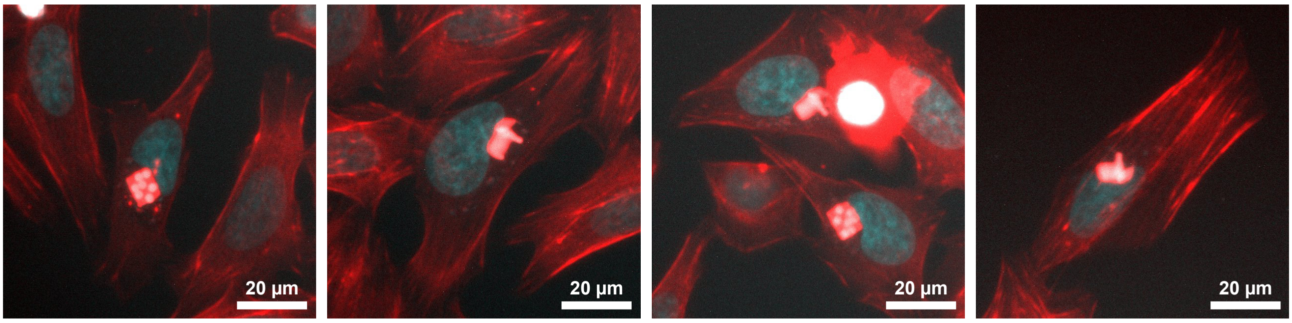

Supplementary Figure 4: *Fluorescence images of actin-stained cells containing printed structures. The actin filaments (red) and nuclei (cyan) can be seen to deform to accommodate the structures (also red). The images are combined from two separately imaged channels and artificially colored. For each channel the contrast is adjusted separately. A time-lapse containing cells in the left three panels is shown in Supplementary Video 3.*

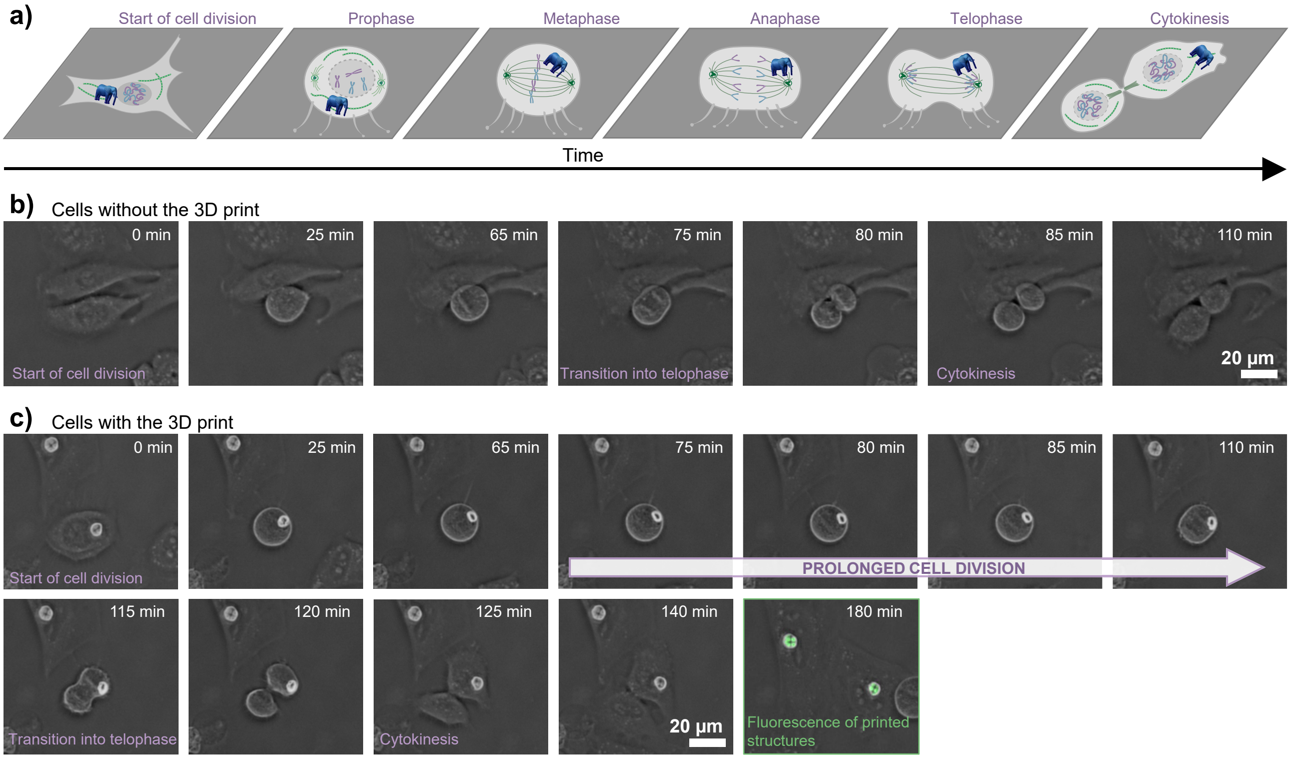

Supplementary Figure 5: *Comparison of the duration of cell division for cells with and without the printed structures inside.* ***(a)*** *Schematics of cell mitosis, where the printed structure remains in one of the daughter cells.* ***(b)*** *A time lapse of mitosis for a non-treated cell.* ***(c)*** *A time lapse of mitosis for a cell containing a printed structure. Snapshots in (b) and (c) are inverted bright field (BF) images.*

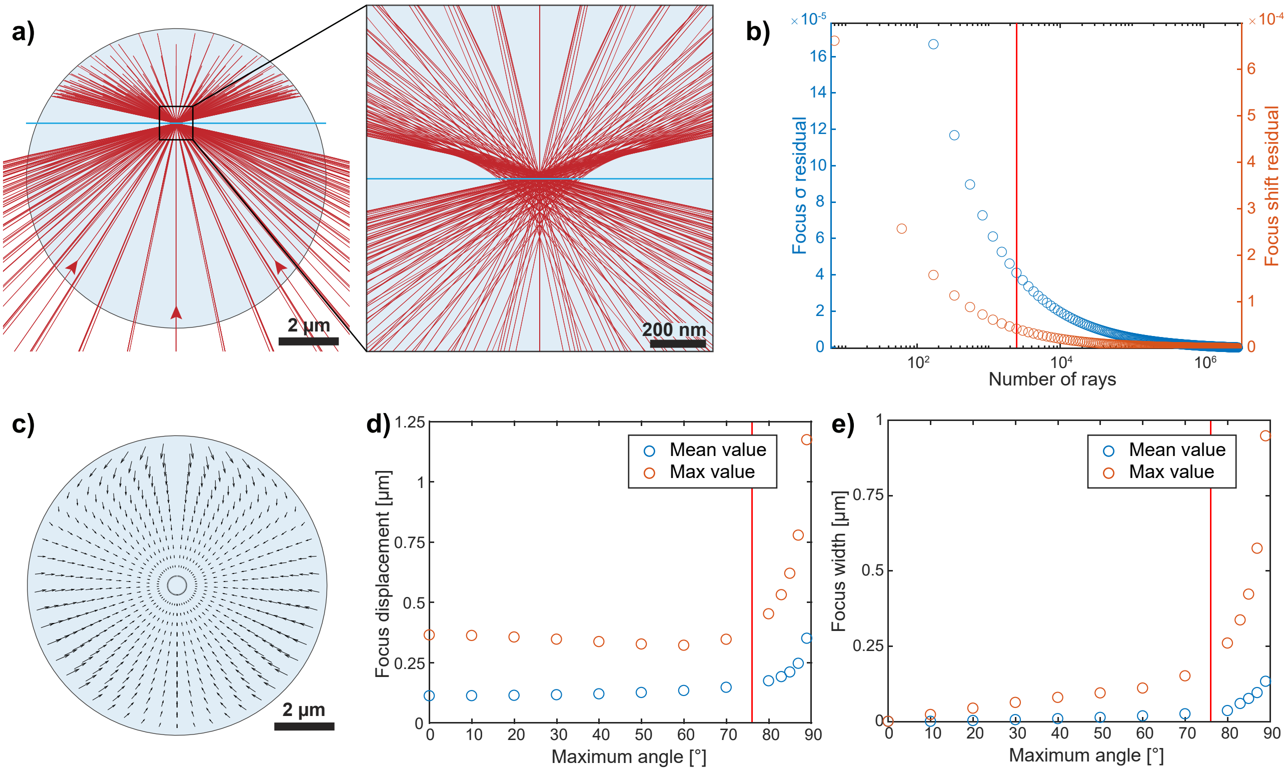

Supplementary Figure 6: *Additional results from the simulation of printing in a droplet.* ***(a)*** *Rays, refracted at the droplet surface, as obtained in a 3D simultion, and the zoom-in of the image. Instead of all the rays meeting in a single spot, due to refraction, defocusing occurs. Image shows a projection of all the rays (in 3D) to the meridian plane of the droplet.* ***(b)*** *Residuals of defocusing and focus displacement in dependence of the number of rays. Residuals are defined as the difference in the result compared to the result obtained with* $3 \cdot 10^6$ *rays. The vertical line marks the number of rays used in the simulation (2437). As can be seen from the plot, this is sufficient to obtain the displacement and width of the focus with the accuracy higher than* $5 \cdot 10^{-5}$ *droplet size.* ***(c)*** *Direction of the focus displacement is shown here as an addition to Figure 3b, where only absolute values of the focus displacements are shown. In the droplet interior, the displacements are small and oriented radially towards the center, whereas at the droplet edges their direction can vary substantially.* ***(d)*** *Dependence of the focus displacement on the maximum angle of rays entering the droplet. Only results for the bottom hemisphere are shown, as on the top half of the droplet the results diverge close to the edge. For obtaining results in Figure 3b,c the maximum angle was calculated based on the objective characteristics and refractive indices of the materials used in the experiment (marked here with a red line).* ***(e)*** *Dependence of the defocusing on the maximum angle of rays entering the droplet (only bottom hemisphere). It can be seen that both values are only weakly affected by the increase in the entrance angle. The NA of the objective and refractive indices of media used in this study result in the entrance angle of 76 ° (red line), which is close to the optimum considering that with lower NA the diffraction is increased.*

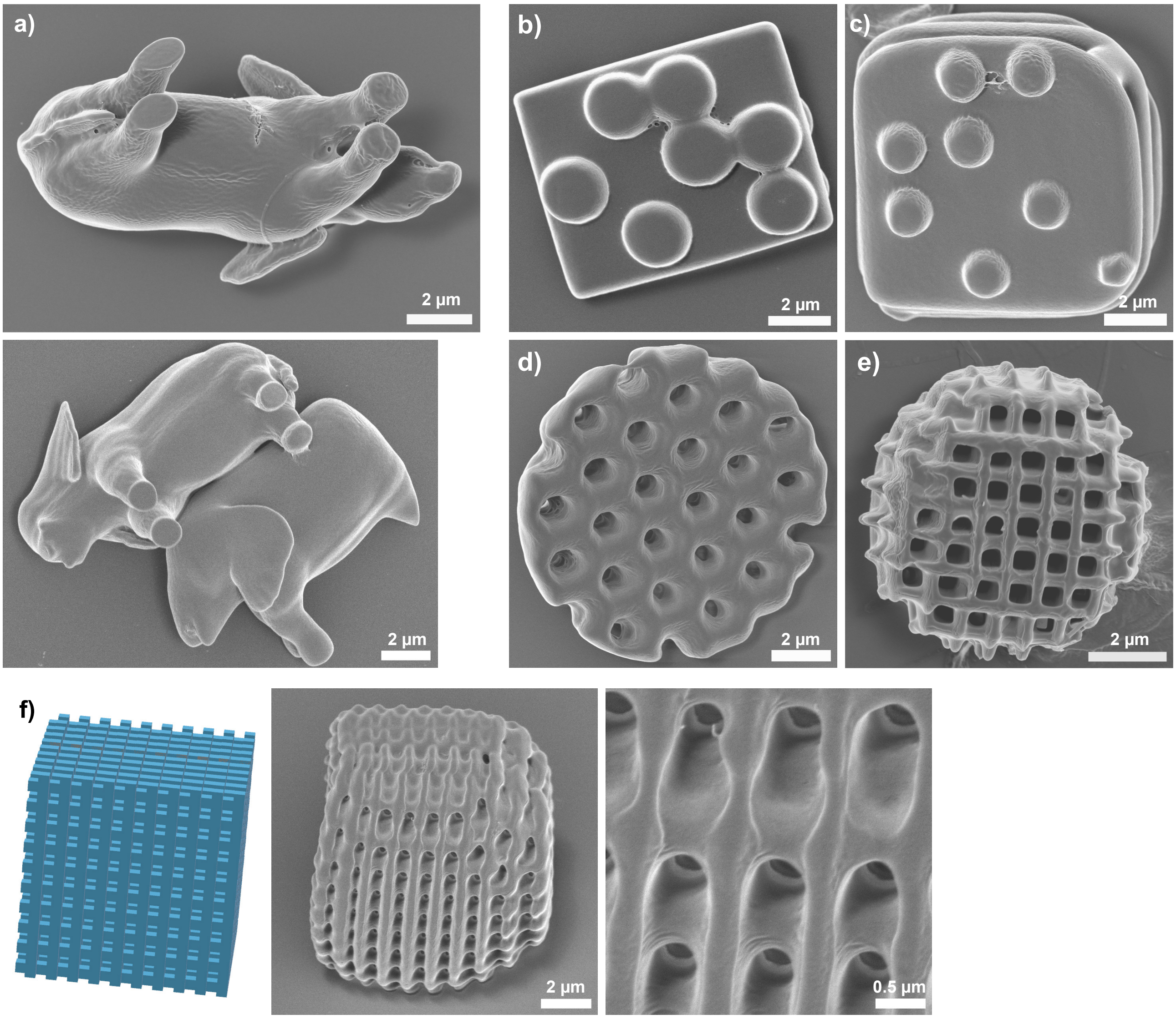

Supplementary Figure 7: *SEM images of various structures printed in droplets of photoresist in an aqueous environment.* ***(a)*** *Elephants, printed by the same design as in Figure 1d,e, and Figure 3g,h,i, and viewed from different angles.* ***(b)*** *Logo of J. Stefan Institute, printed by the same design (Figure 2b) as in Figure 2a,c,d. Here, the structure was printed in a larger droplet, allowing the entire design to be printed.* ***(c)*** *A 3D barcode printed by the design shown in Figure 4a.* ***(d)*** *A hexagonal diffraction grating printed by the design shown in Figure 4b.* ***(e)*** *A zoomed-out image of the structure in Figure 3e, printed by the design shown in Figure 3d. This structure was printed in a smaller droplet as the one in Figure 3d, therefore here less of the cubic-grid design got printed.* ***(f)*** *A different cubic-grid 3D design, a printed structure and its zoom-in.*

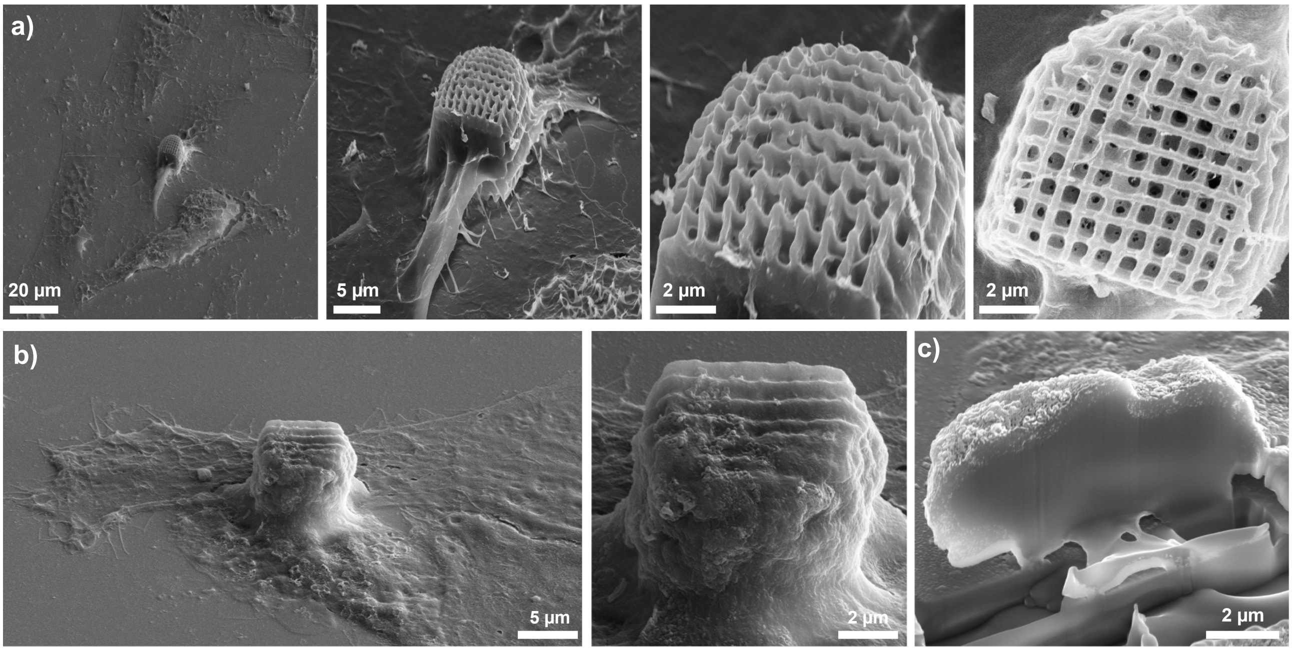

Supplementary Figure 8: *SEM images of cells containing structures printed inside.* ***(a)*** *A woodpile structure by the design in Figure 3d, printed inside a cell. During the preparation of the sample for SEM imaging, the cells were accidentally dried up too much, so they were completely flattened on the substrate. However, this made the printed structure very well-visible, confirming the high fidelity of the prints also in cells.* ***(b)*** *In a sample, where cells were more optimally preserved for SEM imaging, the woodpile structure's positioning inside the cell is even more obvious.* ***(c)*** *An additional cross-section of the elephant structure in Figure 3h,i, obtain by cutting with a focused ion beam. The cell material on top of the structure in inhomogeneous, whereas the printed structure appears homogeneous and non-porous.*

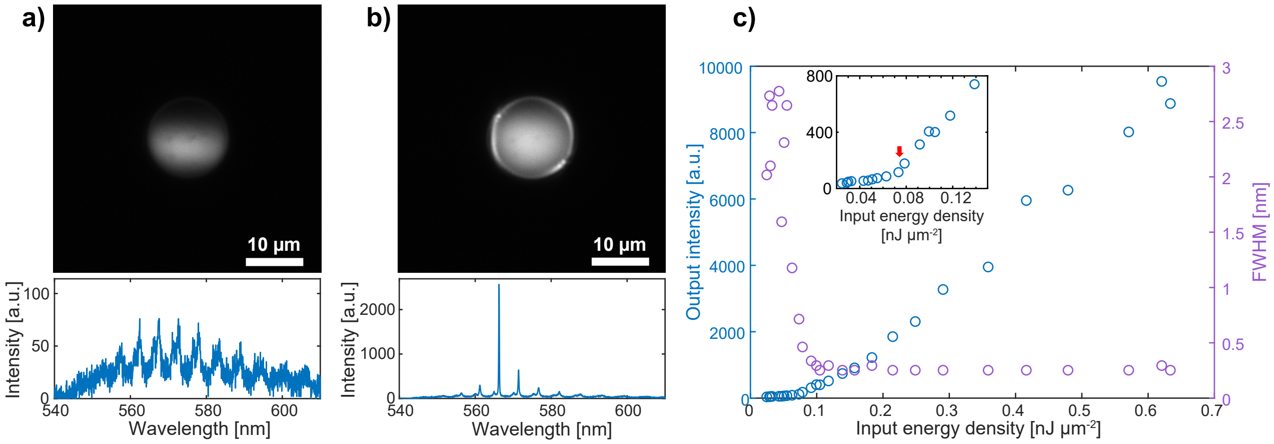

Supplementary Figure 9: *Characterization of a WGM laser, made from a high-refractive-index photoresist dyed with a fluorescent dye. This characterization was performed on a microlaser in a water environment, similar to the one shown in Figure 4i-k in the main text.* ***(a)*** *Below the lasing threshold fluorescence is excited inside the microcavity. In the emission spectrum WGM resonances can be observed.* ***(b)*** *At higher pump energies, a bright speckle pattern indicative of lasing can be seen at the droplet edge. Sharp laser lines appear in the spectrum.* ***(c)*** *Dependence of the output intensity and FWHM of the most prominent peak in the spectrum on the increasing energy density of the pump laser. An inset shows a zoomed-in plot of the output intensity vs. pump energy density, where a typical two-slope lasing curve can be observed. The arrow indicates a lasing threshold of approximately 0.07 nJ* μm^{-2}.

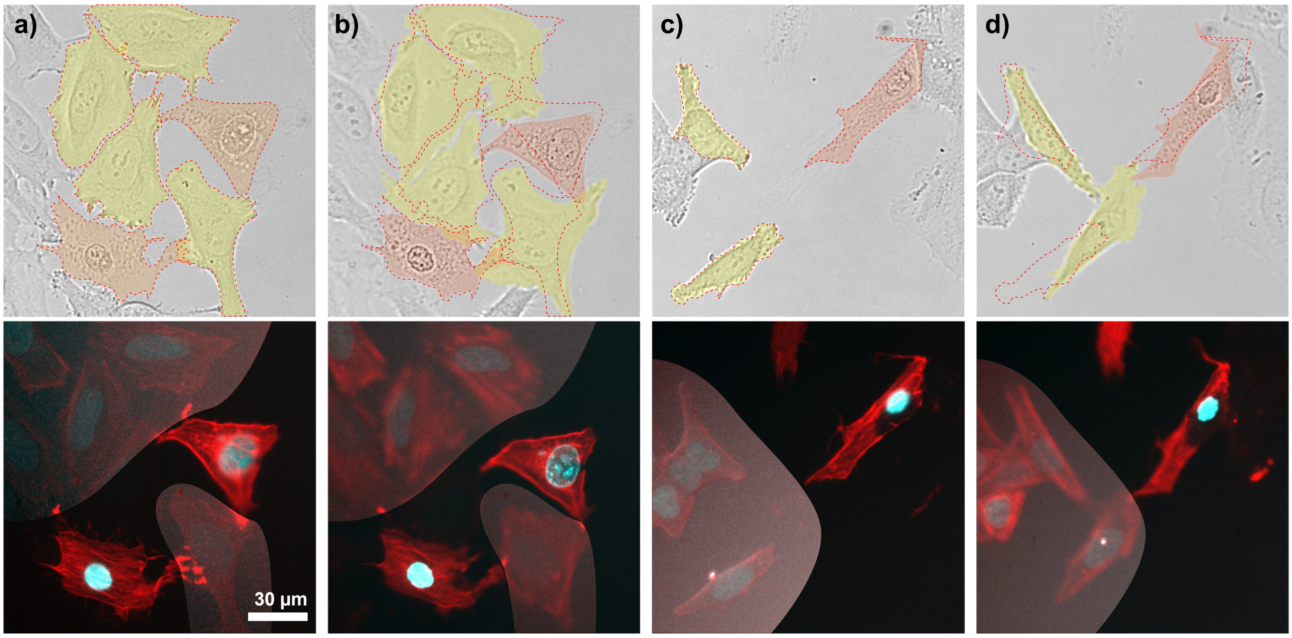

Supplementary Figure 10: *Cell fixation with a water-soluble hydrogel photoresist, imaged in bright-field (top) and in fluorescence imaging mode (bottom).* ***(a)*** *Cells, injected with the hydrogel photoresist (shaded orange) and control-ones, that haven't been manipulated (shaded yellow), imaged 40 min after injections being finished. In the bottom panel the injected cells appear very bright in fluorescence. To see the control-ones, contrast had to be severely increased in the blue-outlined parts of the images.* ***(b)*** *Same cells after 2 hours. The red dashed line shows the outline of the cells after the injection (panel a). The cells without injected ink (yellow) visibly change in shape, whereas the injected ones (orange) stay fixed. Also in fluorescence, the actin microfilaments (red) appear fixed.* ***(c)*** *Hydrogel photoresist is injected into a cell (shaded orange) and illuminated with a CW 404 nm laser.* ***(d)*** *Two hours later, the injected cell's shape remains the same, whereas the other cells (yellow) have moved and changed in shape. Same can be seen in the bottom fluorescence panels. In blue-outlined parts of the images the contrast is severely increased. All fluorescent images are artificially colored.*

Supplementary Discussion

Indication of increased cell mobility after mitosis for cells containing 3D printed structures

We observed that, after cell division, particular cells containing a printed structure show significant change in the mobility and cell elasticity/deformability (Supplementary Video 4). This could indicate another interesting biological effect that can be related to the large 3D printed structures inside cells. In the Supplementary Video 4, the tracks and shapes of individual cells are color-coded according to the cell displacement. Only the cell with the printed structure experiences an increase in mobility after the cell division (yellow and red tracks). The quantification of the cell mobility was performed by a Cellpose algorithm [1], providing automated training, detection, segmentation and tracking. The individual track is obtained from the positions of the center of mass in time for the individual segmented cell. Similar behavior of increased mobility after the cell division was observed in several other cells containing the structures, but not all. This is a preliminary observation that the structures within the cells could be used to change the behavior and possibly even the phenotype of the cells.

Preliminary results with a water-soluble hydrogel photoresist in cells

When printing in a droplet of photoresist inside a cell, printing is limited to the droplet volume. For example, printing a cage around an organelle or fixing a certain part of the cell by polymerizing it is not possible in this approach. To come closer to this goal, we did preliminary experiments using a water-soluble hydrogel photoresist. We used a photo-responsive hydrogel ink, HydroBioINX N400 (by Bioinx, Belgium), which is based on gelatine and was developed for 3D bioprinting with a bioink containing cells. To act as a bioink, the resin is mixed with a proprietary crosslinker, previously dissolved in a proprietary buffer. This bioink is typically used as a substitute for an extracellular matrix. In the current experiment, where the resin was injected into a cell and mixed with the cell contents, we used a slightly modified protocol for resin preparation. After heating all the components (resin, cross-linker, buffer) to 37 °C, a vial of cross-linker was filled with 0.5 ml of the buffer. 10 µl of this solution was added into 90 µl of the resin. The mixture was filled into a micropipette tip for the injection. Due to the fact, that the resin gelates at decreased temperature, the microinjection was performed at 37 °C. The cells were submerged in 1 ml of DMEM which acted as a heat reservoir for the micropipette. The injection pressure was set to 1000 hPa and the injection time was 4 s. If the cell was to be illuminated, the illumination with a violet continuous-wave laser (404 nm) took place right after the injection. After all the cells in the experiment were injected, NucSpot Live 488 (by Biotium, USA) and SPY555-actin dye (by Spirochrome) were added to the cell culture medium to label cell nuclei and actin microfilaments, respectively.

Supplementary Figure 10a shows cells $\sim$ 40 min after the injections were finished. The cells that were injected with the hydrogel photoresist are shaded orange in the top panel. In the bottom one, we can see that the injected cells accumulated a large quantity of dyes, appearing very bright in fluorescence images. In parts of the image with non-manipulated cells, the contrast and brightness had to be increased, to see them, but they still appear very dark. Supplementary Figure 10b shows the situation 2 hours later, where the red outlines mark the starting shape of the cells. Supplementary Figure 10c shows a cell (shaded orange), that was injected with the hydrogel photoresist and illuminated with a 404 nm CW laser shortly after, and Supplementary Figure 10d the situation after 2 hours. It can be seen that the cells injected with hydrogel photoresist become rigid, regardless of whether they get illuminated (Supplementary Figure 10c,d) or not (Supplementary Figure 10a,b). This can be useful to fix entire cells in the presence of non-fixed viable cells, for example to study certain diseases

that cause changes in tissue stiffness. However, to further advance this approach, selective polymerization of only specific parts of the cell is needed. We expect this can be achieved by preforming a systematic scan over the cross-linker/resin ratio and by changing the injected volume with respect to the cell volume to find appropriate parameters that would yield a polymerized structure only where laser illumination took place within the cell. While in the current experiments the cells die due to the overall fixation (which can be seen by an excessive dye load within), we expect that selective polymerization of only small regions within the cells would not kill the cells instantly, thus enabling studying of different cell processes and organelle functioning.

Supplementary video legends

Supplementary Video 1:

Behavior of a cell, containing a 3D-printed structure. A sped-up (6000x speed) behavior of a HeLa cell containing a 3D-printed structure in the shape of an IJS logo over 1 day, imaged in fluorescence. The cell nuclei are labeled with NucSpot Live 488 (by Biotium, USA) and the cell membranes with CellMask Orange dye (by Invitrogen, USA). The movie starts approximately two hours after printing, when the samples had been successfully labeled. Scale bar 20 µm, time format hh:mm.

Supplementary Video 2:

Cell division. Three sped-up videos (1500x speed), each show a HeLa cell containing a 3D-printed structure undergoing cell division. Scale bar 10 µm. Animated version of Figure 2a.

Supplementary Video 3:

Actin-stained cells containing structures. A sped-up time-lapse movie (3000x speed) shows structures in live cells. Actin filaments are stained with SPY555-actin dye (Spirochrome) and cell nuclei with NucSpot Live 488 (Biotium, USA). Images were taken with a monochrome camera in two separate channels and colored artificially. The contrast was adjusted for each channel separately. Time format hh:mm.

Supplementary Video 4:

Increased cell mobility after mitosis. Automated cell tracking in a time-lapse experiment. Centers of mass for each cell position in time are connected with straight lines. Daughter cells originating from the cell in the center, which contains a 3D printed structure, have a much higher mobility than the original cell, as well as larger mobility than cells without the printed objects. Trajectories of the cells are color-coded to show the total cell displacement in µm (colorbar at the bottom). Time format hh:mm.

Supplementary Video 5:

Rigidity of the structures. Two sped-up videos (3600x speed), each showing a HeLa cell containing a 3D-printed elephant structure undergoing cell division. In the first video, a spherical structure resides in the same cell as the elephant. While the elephant structures are being pulled around the cells, they remain rigid and even the thin trunks do not exhibit visible deformations. Scale bar 20 µm. The first video is an animated version of Supplementary Figure 3c.

Supplementary Video 6:

3D-printing inside cells. Three real-time videos of photo-polymerization taking place in droplets of IP-S photoresist injected into HeLa cells. The first one shows printing of a 3D barcode (Figure 4a,b), the second one, printing of a hexagonal diffraction grating (Figure 4c) and the third one, printing of an elephant (such as in Figure 1d-f and Figure 3h,i). During printing, the laser beam follows a pre-defined path to illuminate a desired pattern, resulting in photo-polymerization. In-plane beam-stirring is performed by galvo-mirrors. After illuminating one plane, the sample is translated via piezo stage along the z-axis, and printing of the next plane starts.

Supplementary References

[1] Carsen Stringer, Tim Wang, Michalis Michaelos, and Marius Pachitariu. Cellpose: a generalist algorithm for cellular segmentation. *Nature Methods*, 18(1):100–106, 2021.
